# Association between *Helicobacter pylori* infection and serum uric acid levels in a Chinese community population: a cross-sectional study stratified by renal function

**DOI:** 10.3389/fmed.2025.1615161

**Published:** 2025-08-29

**Authors:** Keding Wang, Jiaojiao Zheng, Shali Yin, Hui Zhang, Hongying Yan, Yuling Jin, Yuzhen Qiu, Xvru Wang, Xinyu Zhu, Lan Yao, Shenglan Tian

**Affiliations:** ^1^Department of General Medicine, Tianyou Hospital Affiliated to Wuhan University of Science and Technology, Wuhan, China; ^2^School of Clinical Medicine, Wuhan University of Science and Technology, Wuhan, China; ^3^Hongshan Street Community Health Service Center Affiliated to Wuhan University of Science and Technology, Wuhan, China

**Keywords:** *Helicobacter pylori*, serum uric acid, renal function, effect modification, Chinese population

## Abstract

**Background:**

The relationship between *Helicobacter pylori* (*H. pylori*) infection and serum uric acid levels remains debated. This study investigates the association between *H. pylori* infection and serum uric acid levels in a Chinese community, exploring renal function as a potential modifier.

**Methods:**

We conducted a cross-sectional study involving 8,439 adults who underwent health examinations at a hospital in Wuhan from January 2022 to January 2024. *H. pylori* infection was assessed via the 14C-urea breath test, and serum uric acid levels were measured by the uricase method. Multivariable linear regression models evaluated the associations, and interaction analysis identified potential effect modifiers. Subgroup analyses were stratified by estimated glomerular filtration rate (eGFR).

**Results:**

The prevalence of *H. pylori* infection was 21.5% (1,816/8,439). Initial analysis showed higher serum uric acid levels in individuals with *H. pylori* infection compared to those without (403.76 ± 102.89 vs. 395.87 ± 102.13 μmol/L, *p* = 0.004). However, after adjusting for age, sex, body mass index, lipid profiles, and hepatorenal function, this association was no longer significant in the overall cohort (*β* = 1.92, 95% CI: −2.38 to 6.23, *p* = 0.381). Interaction analysis revealed a significant modification by eGFR (*p* for interaction = 0.007). Stratified analysis showed an inverse association between *H. pylori* infection and serum uric acid in individuals with mild renal impairment (eGFR 60–80 mL/min/1.73m^2^, *n* = 824; adjusted *β* = −17.86, 95% CI: −31.28 to −4.44, *p* = 0.009), while no such association was observed in those with normal renal function (eGFR ≥80 mL/min/1.73m^2^, *n* = 7,531; *β* = 3.92, 95% CI: −0.66 to 8.50, *p* = 0.094). Sensitivity analyses confirmed the robustness of these findings.

**Conclusion:**

Renal function modulates the association between *H. pylori* infection and serum uric acid levels, with an inverse correlation observed in individuals with mild renal impairment. These findings suggest that renal function may influence the impact of *H. pylori* infection on uric acid metabolism.

## Introduction

*Helicobacter pylori*, a Gram-negative bacterium that colonizes the human gastric mucosa, infects nearly half of the global population, presenting a significant public health concern (1–4). Its prevalence varies across regions, with developing countries exhibiting higher infection rates (5–7). In China, approximately 50% of the population is infected, though regional variations exist due to socioeconomic factors, lifestyle choices, and demographic characteristics (8–12). Since Warren and Marshall first isolated *H. pylori* in 1982, research has shown that its pathogenic effects extend beyond the gastrointestinal tract, contributing to conditions such as peptic ulcers, chronic gastritis, gastric cancer, and even neurodegenerative diseases (13–17).

Recent studies have highlighted that *H. pylori* influences host metabolic homeostasis through multiple mechanisms. For instance, VacA targets mitochondria, leading to Adenosine Triphosphate (ATP) depletion and cellular energy stress, which activates AMP-activated protein kinase (AMPK) and shifts cellular processes from anabolic to catabolic through mechanisms including autophagy induction (18–20). Chronic low-grade inflammation induced by *H. pylori* infection elevates pro-inflammatory cytokines, such as Tumor Necrosis Factor-*α* (TNF-α) and Interleukin-6 (IL-6), impairing insulin signaling and contributing to insulin resistance (21, 22). Additionally, *H. pylori* alters the gut microbiota, affecting short-chain fatty acid production and the secretion of metabolic hormones such as ghrelin and leptin, which in turn influence systemic energy metabolism (23–25). These extra-gastric effects provide a biological basis for linking *H. pylori* infection with metabolic disorders, including disturbances in uric acid metabolism.

Uric acid, the final product of purine metabolism, acts as an antioxidant at normal concentrations by scavenging reactive oxygen species and protecting against oxidative stress (26–28). However, elevated serum uric acid levels are an independent risk factor for metabolic and cardiovascular diseases, including diabetes, hypertension, and metabolic syndrome (29–31). Uric acid homeostasis is closely tied to renal function, with the kidneys playing a central role in its clearance. Approximately 70% of uric acid is cleared by the kidneys through transporters such as Urate Transporter 1 (URAT1) and Glucose Transporter 9 (GLUT9) (32, 33). As renal function declines, reduced renal clearance of uric acid triggers compensatory excretion via intestinal ATP-Binding Cassette Subfamily G Member 2 (ABCG2) transporters (33, 34). This interaction between the kidneys and gut—referred to as the “kidney-gut axis”—suggests that factors such as *H. pylori* infection may affect uric acid metabolism differently depending on renal function, offering a new perspective on the variability seen in past studies (34–36).

The relationship between *H. pylori* infection and serum uric acid levels remains contentious. Some studies suggest that *H. pylori* infection is a risk factor for gout, while others report no significant association (37, 38). This inconsistency may be attributed to the modifying role of renal function. Research has shown that patients with chronic kidney disease (CKD) have lower *H. pylori* infection rates (48.2% vs. 59.3%), and the relationship between uric acid and clinical outcomes varies significantly across different stages of renal function (39, 40). In the early stages of renal decline, uric acid levels are positively correlated with cardiovascular risk, while this association weakens or disappears in later stages (40, 41). Furthermore, *H. pylori*’s impact on renal health appears to be stage-dependent, suggesting that renal function may modulate the interaction between *H. pylori* infection and uric acid metabolism through altered gastrointestinal conditions, inflammatory responses, and the function of uric acid transporters (32, 34, 35, 39).

Given the conflicting evidence, the lack of consideration for potential effect modifiers, and the scarcity of studies in Chinese populations, this study aims to evaluate the relationship between *H. pylori* infection and serum uric acid levels in a large cohort from China, with a specific focus on renal function as a modifying factor. We hypothesize that the association between *H. pylori* infection and serum uric acid levels will vary depending on renal function status, which may help explain the inconsistencies observed in previous studies and offer a theoretical framework for more personalized infection management and metabolic monitoring strategies.

## Methods

### Study design and population

**Figure 1:**
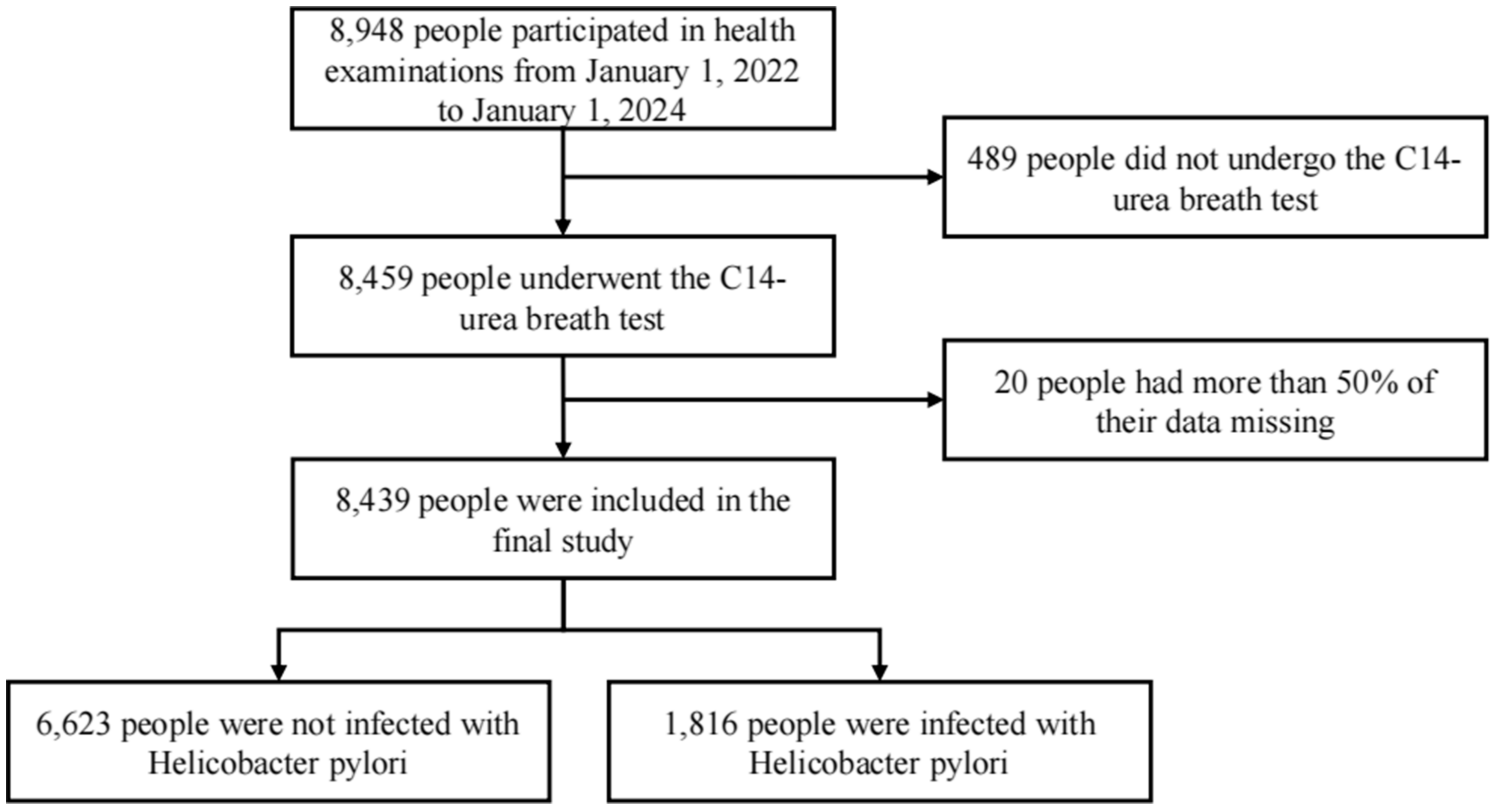
Study flowchart.

This retrospective cross-sectional study utilized health examination data from Tianyou Hospital, affiliated with Wuhan University of Science and Technology, and its community health service centers. Data were collected between January 2022 and January 2024. The study protocol was approved by the Ethics Committee of Tianyou Hospital (approval number: No. LL2024100901), and written informed consent was obtained from all participants. Initially, 8,948 adults (≥18 years) undergoing health examinations were included, predominantly asymptomatic individuals with a small proportion presenting mild, non-specific gastrointestinal symptoms, such as dyspepsia or epigastric discomfort. Inclusion criteria were: (1) completion of the 14C-urea breath test (14C-UBT); (2) availability of complete demographic and blood biochemistry data; (3) no use of proton pump inhibitors, bismuth preparations, antibiotics, or other anti-*H. pylori* medications within 4 weeks prior to testing; (4) voluntary participation with consent for data use. Exclusion criteria included: (1) missing key test data (14C-UBT, demographic data, or blood tests); (2) current or past use of uric acid-lowering medications (e.g., allopurinol, febuxostat, benzbromarone); (3) history of gastric cancer or gastrectomy; (4) missing data rate >50%. After rigorous screening, 8,439 participants were included in the final analysis (Figure 1).

### *Helicobacter pylori* infection detection

*Helicobacter pylori* infection status was assessed using the 14C-UBT. Participants fasted for at least 8 h before the test. The procedure involved ingesting a capsule containing 1.0 μCi of 14C-labeled urea, followed by breath sample collection 15 min later using a GP1000 breath analyzer (Furui Kang, China). A diagnostic threshold of 100 dpm/mmol CO₂ was set, with values ≥100 dpm/mmol CO₂ considered positive for *H. pylori* and values <100 dpm/mmol CO₂ considered negative. This method has demonstrated high diagnostic accuracy, with sensitivity and specificity exceeding 95% (42).

### Serum uric acid measurement

Venous blood samples were collected after a minimum 8-h fasting period. Serum uric acid concentrations were measured using the uricase-peroxidase method on an ADVIA 2400 automatic biochemistry analyzer (Siemens, Germany). The enzymatic method converts uric acid to allantoin and hydrogen peroxide, which then reacts with chromogenic substrates for colorimetric quantification. The detection range was 0–20 mg/dL (0–1,190 μmol/L), with intra-assay and inter-assay coefficients of variation (CV) of <2 and <3%, respectively. Hyperuricemia was defined as serum uric acid >420 μmol/L in males and >360 μmol/L in females (43).

### Covariate collection and measurement

Covariates were selected based on existing literature and included age, sex, body mass index (BMI), mean arterial pressure (MAP), smoking status, alcohol consumption, occupational activity, hypertension, diabetes, and various laboratory parameters. Age and sex were obtained via structured questionnaires. BMI was measured using a standardized electronic height-weight scale (MSG005-H, Meilun Medical, China), with BMI calculated as weight (kg)/height^2^ (m^2^). Blood pressure was measured according to American Heart Association guidelines, with the average of three measurements taken after a 5–10 min rest. MAP was calculated using the formula: MAP = diastolic pressure + (systolic pressure - diastolic pressure)/3. Smoking and alcohol consumption were classified into three categories: (1) never exposed; (2) former exposure (≥1 year cessation); (3) current exposure (cessation <1 year). Occupational intensity was assessed using metabolic equivalents (METs) based on the Compendium of Physical Activities (44). Participants reported their occupational type and the activity composition of a typical workday. Corresponding METs values were assigned to each activity, and participants were classified into three categories: light (<3.0 METs, primarily sedentary work, such as office workers); moderate (3.0–6.0 METs, involving frequent standing or moderate physical activity, such as sales staff and nurses); and heavy (>6.0 METs, involving continuous heavy physical labor, such as construction workers and porters). Hypertension was defined as: (1) prior physician diagnosis; (2) current antihypertensive medication; (3) systolic ≥140 mmHg or diastolic ≥90 mmHg (based on ≥2 measurements). Diabetes was defined as: (1) glucose-lowering medications; (2) fasting glucose ≥7.0 mmol/L (based on ≥2 measurements); or fasting glucose between 6.1–6.9 mmol/L (considered as diabetes for binary analysis). Blood samples were analyzed for lipid profile, liver function markers (alanine aminotransferase, aspartate aminotransferase, bilirubin), fasting glucose, and serum creatinine. Renal function was evaluated using the estimated glomerular filtration rate (eGFR), calculated via the Chronic Kidney Disease Epidemiology Collaboration (CKD-EPI) equation. A complete blood count was measured using standard methods. All tests were performed within 4 h of blood collection, and laboratory procedures adhered to National Center for Clinical Laboratory quality control standards.

### Statistical analysis

Continuous variables were expressed as means ± standard deviation, and categorical variables as frequencies (percentages). Baseline characteristics were compared between *H. pylori*-positive and negative groups using one-way Analysis of Variance (ANOVA) for continuous variables and chi-square tests for categorical variables. Linear regression assumptions were tested, and multicollinearity was assessed using variance inflation factors (VIF), excluding variables with VIF > 5. The association between *H. pylori* infection and serum uric acid was analyzed using multivariable linear regression in three hierarchical models: Model 1 (unadjusted), Model 2 (adjusted for age and sex), and Model 3 (fully adjusted for all covariates). Interaction analysis was conducted to identify effect modifiers by including interaction terms between *H. pylori* infection and potential modifiers, with likelihood ratio tests used to assess statistical significance (*p* < 0.05). For continuous effect modifiers showing significant interactions, grid search algorithms were applied to determine optimal stratification thresholds based on model fit criteria. Sensitivity analyses included logistic regression with hyperuricemia as a binary outcome and linear regression using log-transformed uric acid values to account for non-normality. All analyses were performed using R version 4.5.1, with two-sided *p*-values <0.05 considered statistically significant.

## Results

### Characteristics of the study population

A total of 8,439 participants were included in the study, of whom 6,623 (78.48%) were *H. pylori*-negative and 1,816 (21.52%) were *H. pylori*-positive. The *H. pylori*-positive group had a higher proportion of males (80.84% vs. 77.65%, *p* = 0.004), current smokers (37.06% vs. 32.09%, *p* < 0.001), current drinkers (35.35% vs. 32.90%, *p* = 0.036), and individuals engaged in heavy physical labor (45.48% vs. 40.45%, *p* = 0.001). Biochemically, the *H. pylori*-positive group exhibited significantly higher mean arterial pressure (MAP) (94.02 ± 13.69 vs. 92.87 ± 12.64 mmHg, *p* = 0.001), white blood cell count (6.60 ± 1.68 vs. 6.28 ± 1.58 × 10^9^/L, *p* < 0.001), and platelet count (246.55 ± 62.27 vs. 241.92 ± 57.80 × 10^9^/L, *p* = 0.003). Conversely, high-density lipoprotein cholesterol (HDL-C) levels were significantly lower in the *H. pylori*-positive group (1.33 ± 0.33 vs. 1.37 ± 0.34 mmol/L, *p* < 0.001). Serum uric acid levels were also higher in the *H. pylori*-positive group compared to the negative group (403.76 ± 102.89 vs. 395.87 ± 102.13 μmol/L, *p* = 0.004) (Table 1).

**Table 1:** Characteristics of study participants at baseline.

Variables	*H. pylori* Negative (*n* = 6,623)	*H. pylori* Positive (*n* = 1816)	*p*-value
Sex, %			
Female	1,480 (22.35%)	348 (19.16%)	0.004
Male	5,143 (77.65%)	1,468 (80.84%)	
Drinking, %			
Never	3,100 (46.81%)	789 (43.45%)	0.036
Former	1,344 (20.29%)	385 (21.20%)	
Current	2,179 (32.90%)	642 (35.35%)	
Smoking, %			
Never	3,137 (47.37%)	758 (41.74%)	<0.001
Former	1,361 (20.55%)	385 (21.20%)	
Current	2,125 (32.09%)	673 (37.06%)	
Occupational intensity, %			
Light	756 (11.41%)	195 (10.74%)	0.001
Moderate	3,188 (48.14%)	795 (43.78%)	
Heavy	2,679 (40.45%)	826 (45.48%)	
Hypertension, %			
No	4,757 (71.83%)	1,262 (69.49%)	0.055
Yes	1866 (28.17%)	554 (30.51%)	
Diabetes, %			
No	5,706 (86.15%)	1,549 (85.30%)	0.372
Yes	917 (13.85%)	267 (14.70%)	
Age (years)	43.85 ± 12.42	44.14 ± 12.06	0.372
BMI (kg/m^2^)	24.67 ± 3.53	24.81 ± 3.57	0.139
MAP (mmHg)	92.87 ± 12.64	94.02 ± 13.69	0.001
HDL-C (mmol/L)	1.37 ± 0.34	1.33 ± 0.33	<0.001
LDL-C (mmol/L)	2.95 ± 0.77	3.01 ± 0.76	0.01
TC (mmol/L)	4.82 ± 0.93	4.85 ± 0.91	0.226
TG (mmol/L)	1.86 ± 1.88	1.90 ± 1.72	0.415
ALT (U/L)	29.39 ± 22.72	29.57 ± 22.72	0.76
AST (U/L)	23.49 ± 12.40	23.82 ± 13.58	0.335
TBil (μmol/L)	16.44 ± 6.23	16.13 ± 6.00	0.057
DBil (μmol/L)	6.11 ± 2.23	6.03 ± 2.08	0.156
eGFR (mL/min/1.73 m^2^)	101.69 ± 18.92	101.46 ± 19.56	0.642
UA (μmol/L)	395.87 ± 102.13	403.76 ± 102.89	0.004
FBG (mmol/L)	5.35 ± 1.36	5.41 ± 1.48	0.067
RBC (10^12^/L)	4.89 ± 0.47	4.90 ± 0.48	0.292
WBC (10^9^/L)	6.28 ± 1.58	6.60 ± 1.68	<0.001
PLT (10^9^/L)	241.92 ± 57.80	246.55 ± 62.27	0.003

*H. pylori, Helicobacter pylori*; BMI, BodyMass Index; MAP, Mean Arterial Pressure; HDL-C, High-Density Lipoprotein Cholesterol; LDL-C, Low-Density Lipoprotein Cholesterol; TC, Total Cholesterol; TG, Triglycerides; ALT, Alanine Aminotransferase; AST, Aspartate Aminotransferase; TBil, Total Bilirubin; DBil, Direct Bilirubin; eGRF, estimated Glomerular Filtration Rate; UA, Serum Uric Acid; FBG, Fasting Blood Glucose; RBC, Red Blood Cell count; WBC, White Blood Cell count; PLT, Platelet count.

### Association between *Helicobacter pylori* infection and serum uric acid

In the unadjusted model (Model 1), *H. pylori* infection was positively associated with serum uric acid levels (*β* = 7.89, 95% CI: 2.58 to 13.20, *p* = 0.004). However, after adjusting for confounders, this association weakened. In the age- and sex-adjusted model (Model 2), the association became marginally significant (*β* = 4.39, 95% CI: −0.27 to 9.05, *p* = 0.065). In the fully adjusted model (Model 3), which accounted for metabolic parameters, the association was no longer significant (*β* = 1.92, 95% CI: −2.38 to 6.23, *p* = 0.381) (Table 2).

**Table 2:** Association between *Helicobacter pylori* infection and serum uric acid levels using hierarchical linear regression models.

Variable	*n* (%)	Model 1		Model 2		Model 3	
		*β* (95% CI)	*p*-value	*β* (95% CI)	*p*-value	*β* (95% CI)	*p*-value
*H. Pylori*							
Negative	6,623 (78.48)	Reference	0.004	Reference	0.065	Reference	0.381
Positive	1816 (21.52)	7.89 (2.58, 13.20)		4.39 (−0.27, 9.05)		1.92 (−2.38, 6.23)	

Model 1: Unadjusted; Model 2: Adjusted for age and sex; Model 3: Adjusted for all covariates; *β*: regression coefficient representing the difference in serum uric acid between *H. pylori* positive and negative groups; CI: Confidence Interval; Total cholesterol and direct bilirubin were excluded due to multicollinearity (VIF >5).

### Identification of effect modifiers

Interaction analysis revealed that estimated glomerular filtration rate (eGFR) significantly modified the relationship between *H. pylori* infection and serum uric acid (P for interaction = 0.007). Alanine aminotransferase (ALT) also showed a significant interaction (*p* = 0.035). However, traditional effect modifiers such as sex, age, and body mass index (BMI) did not exhibit significant interactions (Supplementary Table 1). A systematic breakpoint analysis identified that the association between *H. pylori* infection and serum uric acid was most significant at eGFR = 85 mL/min/1.73m^2^ (*p* = 0.002). Based on clinical relevance and statistical considerations, a cut-off of 80 mL/min/1.73m^2^ was selected for the primary analysis (*p* = 0.006) (Supplementary Table 2).

### Stratified analysis by renal function

Stratified analysis revealed heterogeneity in the effect of *H. pylori* infection on serum uric acid across different renal function groups. In the mild renal impairment group (eGFR 60–80 mL/min/1.73m^2^, *n* = 824), *H. pylori* infection was inversely associated with serum uric acid levels (*β* = −17.86, 95% CI: −31.28 to −4.44, *p* = 0.009). In contrast, in the normal renal function group (eGFR ≥ 80 mL/min/1.73m^2^, *n* = 7,531), the association was positive but not statistically significant (*β* = 3.92, 95% CI: −0.66 to 8.50, *p* = 0.094). In the severe renal impairment group (eGFR < 60 mL/min/1.73m^2^, *n* = 84), no significant association was observed, likely due to the small sample size (*β* = 25.9, 95% CI: −18.36 to 70.17, *p* = 0.256) (Table 3).

**Table 3:** Association between *Helicobacter pylori* infection and serum uric acid stratified by renal function.

Renal function category	*N*	*β* (95% CI)ᵃ	*p*-value
Severe impairment (eGFR <60 mL/min/1.73m^2^)	84	25.90 (−18.36, 70.17)	0.256
Mild impairment (eGFR 60–80 mL/min/1.73m^2^)	824	−17.86 (−31.28, −4.44)	0.009
Normal function (eGFR ≥80 mL/min/1.73m^2^)	7,531	3.92 (−0.66, 8.50)	0.094

CI, confidence interval; eGFR, estimated glomerular filtration rate.

a, *β* represent the difference in serum uric acid levels between *H. pylori*-positive and *H. pylori*-negative participants. Models were adjusted for age, sex, body mass index, mean arterial pressure, smoking, drinking, occupational intensity, hypertension, diabetes, high-density lipoprotein cholesterol, low-density lipoprotein cholesterol, triglycerides, alanine aminotransferase, aspartate aminotransferase, total bilirubin, estimated glomerular filtration rate, fasting blood glucose, red blood cell count, white blood cell count and platelet count. Total cholesterol and direct bilirubin were excluded due to multicollinearity (VIF >5).

### Sensitivity analyses

Several sensitivity analyses were conducted to verify the robustness of the findings. First, logistic regression with hyperuricemia as a binary outcome revealed that in the mild renal impairment group (eGFR 60–80 mL/min/1.73m^2^, *n* = 824), *H. pylori* infection was associated with a 33% reduced risk of hyperuricemia (odds ratio [OR] = 0.673, 95% CI: 0.461 to 0.983, *p* = 0.040) (Table 4). Second, analysis using log-transformed serum uric acid values showed that in the same group, *H. pylori*-positive individuals had 4.43% lower serum uric acid levels compared to *H. pylori*-negative individuals (95% CI: −7.92% to −1.15%, *p* = 0.009) (Table 5). These results from different statistical approaches further support the main findings. Collinearity diagnostics indicated severe multicollinearity for total cholesterol (VIF = 21.3) and direct bilirubin (VIF = 8.9) in the initial model. After excluding these variables, all VIF values were below 3, confirming no multicollinearity issues in the final model. Re-analysis with the adjusted variable set yielded unchanged results (Supplementary Table 3). Linear regression assumption tests further supported the validity of model assumptions (Supplementary Figures 1–4).

**Table 4:** Association between *Helicobacter pylori* infection and hyperuricemia risk stratified by renal function: logistic regression analysis.

Renal function category	Total (*n*)	Hyperuricemia^a^ *n* (%)	OR (95% CI)^b^	*p*-value
Severe impairment eGFR <60 mL/min/1.73m^2^	84	32 (38.1)	6.78 (0.63, 72.76)	0.114
Mild impairment eGFR 60–80 mL/min/1.73m^2^	824	350 (42.5)	0.67 (0.46, 0.98)	0.040
Normal function eGFR ≥80 mL/min/1.73m^2^	7,531	3,125 (41.5)	1.03 (0.91, 1.17)	0.595

CI, confidence interval; eGFR, estimated glomerular filtration rate; OR, odds ratio.

a, Hyperuricemia defined as serum uric acid >420 μmol/L for males and >360 μmol/L for females, according to Chinese guidelines for diagnosis and treatment of hyperuricemia and gout.

b, Odds ratios adjusted for age, sex, body mass index, mean arterial pressure, smoking, drinking, occupational intensity, hypertension, diabetes, high-density lipoprotein cholesterol, low-density lipoprotein cholesterol, triglycerides, alanine aminotransferase, aspartate aminotransferase, total bilirubin, estimated glomerular filtration rate, fasting blood glucose, red blood cell count, white blood cell count and platelet count. Total cholesterol and direct bilirubin were excluded due to multicollinearity (VIF >5).

**Table 5:** Sensitivity analysis: association between *Helicobacter pylori* infection and log-transformed serum uric acid levels stratified by renal function.

Renal function category	Total (*n*)	*β* ^a^	Percent change (%) (95% CI)^b^	*p*-value
Severe impairment eGFR<60 mL/min/1.73m^2^	84	0.063	6.45 (−4.88, 17.37)	0.275
Mild impairment eGFR 60–80 mL/min/1.73m^2^	824	−0.045	−4.43 (−7.92, −1.15)	0.009
Normal function eGFR ≥80 mL/min/1.73m^2^	7,531	0.008	0.83 (−0.35, 2.01)	0.168

CI, confidence interval; eGFR, estimated glomerular filtration rate.

a, *β* represent the difference in log-transformed serum uric acid levels between *H. pylori*-positive and *H. pylori*-negative groups, adjusted for age, sex, body mass index, mean arterial pressure, smoking, drinking, occupational intensity, hypertension, diabetes, high-density lipoprotein cholesterol, low-density lipoprotein cholesterol, triglycerides, alanine aminotransferase, aspartate aminotransferase, total bilirubin, estimated glomerular filtration rate, fasting blood glucose, red blood cell count, white blood cell count and platelet count. Total cholesterol and direct bilirubin were excluded due to multicollinearity (VIF >5).

b, Percent change calculated as (e^β^ – 1) × 100%, representing the relative difference in serum uric acid levels.

Collinearity diagnostics indicated severe multicollinearity for total cholesterol (VIF = 21.3) and direct bilirubin (VIF = 8.9) in the initial model. After excluding these variables, all VIF values were below 3, confirming no multicollinearity issues in the final model. Re-analysis with the adjusted variable set yielded unchanged results (Supplementary Table 3). Linear regression assumption tests further supported the validity of model assumptions (Supplementary Figures 1–4).

## Discussion

In this cross-sectional study of 8,439 Chinese adults, we found that the association between *H. pylori* infection and serum uric acid levels varied significantly by renal function. Although unadjusted analyses suggested a positive correlation, the association lost statistical significance after adjustment for demographic, metabolic, and hepatorenal covariates indicating that the observed relationship in the general population may reflect shared risk factors rather than a direct effect. Notably, stratified analyses identified a significant inverse association between *H. pylori* infection and serum uric acid levels among individuals with mild renal impairment (eGFR 60–80 mL/min/1.73 m^2^), which remained robust after multivariable adjustment. These findings suggest that renal function modulates the systemic metabolic effects of *H. pylori* infection. However, due to the cross-sectional design, causal inference and temporal directionality cannot be established.

The relationship between *H. pylori* and uric acid metabolism has been inconsistently reported in previous studies. Data from the U. S. National Health and Nutrition Examination Survey (NHANES) demonstrated a positive association, with *H. pylori* positive individuals exhibiting elevated uric acid to HDL cholesterol ratios (OR = 1.15; 95% CI: 1.02 to 1.30; *p* = 0.020) (45). In contrast, a large Chinese health screening cohort (*n* = 76,749) reported significantly lower serum uric acid concentrations among infected individuals (*p* < 0.001) (46). These conflicting findings remain unresolved. Our results provide a potential explanation by identifying renal function as an effect modifier. Variability in eGFR distribution across cohorts may have contributed to the discrepancies. Importantly, neither of the prior studies stratified participants by renal function, which may have masked subgroup specific associations. Our finding that *H. pylori* infection is inversely associated with serum uric acid only in those with mildly reduced eGFR highlights the critical role of organ-specific functional status in modulating systemic consequences of localized infections. This insight underscores the importance of renal stratification in metabolic epidemiology, particularly in studies of chronic infectious exposures.

The observed inverse association in individuals with mild renal impairment warrants mechanistic consideration. We hypothesize that this finding reflects an interaction between *H. pylori* induced metabolic changes and compensatory extra-renal uric acid elimination, which becomes physiologically relevant as renal clearance begins to decline. Chronic *H. pylori* colonization induces low-grade systemic inflammation, characterized by increased levels of IL-1β, IL-6, and TNF-*α* (47, 48). These cytokines upregulate xanthine oxidoreductase (XOR) activity in gastric and intestinal epithelia, enhancing both uric acid production and reactive oxygen species (49–51). Concurrently, inflammatory signaling upregulates intestinal urate transporters particularly ATP-binding cassette subfamily G member 2 (ABCG2) on enterocyte apical membranes, promoting urate excretion into the gut lumen (47, 52). In addition, *H. pylori* urease activity raises local ammonia concentrations and alters pH, potentially enhancing the expression and function of these transporters (53, 54). In individuals with normal renal function (eGFR >90 mL/min/1.73 m^2^), such intestinal adaptations are likely of limited systemic consequence, as renal clearance responsible for 70% of urate elimination remains efficient (55). However, when eGFR declines into the 60–80 mL/min/1.73 m^2^ range, modest reductions in renal excretory capacity may render these intestinal compensatory mechanisms biologically significant. In this setting, *H. pylori* driven inflammatory and transporter mediated effects may enhance gut-renal axis activity, leading to a net reduction in serum uric acid levels (34, 52). This hypothesis is supported by the specificity of our finding to this eGFR category, where impairment is sufficient to trigger compensation but not yet severe enough to overwhelm it. Despite statistical significance, the absolute magnitude of urate reduction observed was modest and unlikely to influence clinical decision-making thresholds for pharmacological intervention, which typically begin at 360–420 μmol/L. Nevertheless, the biological relevance remains compelling. Our findings reveal a previously unrecognized interaction between gastrointestinal infection and uric acid homeostasis, particularly under conditions of early renal dysfunction. The renal function–dependent gradient observed in our stratified analyses further supports this interpretation. Among participants with normal renal function (eGFR ≥80 mL/min/1.73 m^2^), no significant association was detected. In contrast, a clear inverse relationship emerged in the mildly impaired group. Among those with severe renal dysfunction (eGFR <60 mL/min/1.73 m^2^), no association was found (*β* = 25.90; *p* = 0.256), and the wide confidence interval (−18.36 to 70.17) suggests substantial imprecision. Several explanations may account for this null finding. The subgroup sample size was limited (*n* = 84), with a relatively low prevalence of *H. pylori* infection, reducing statistical power. Furthermore, advanced CKD is associated with elevated systemic urea, altered gastric acid profiles, and frequent antibiotic exposure factors that may suppress *H. pylori* colonization. At this stage of renal decline, uric acid homeostasis is often severely dysregulated due to impaired excretion, transporter dysfunction, and polypharmacy (56–58). Thus, the compensatory mechanisms operative in early CKD may be either saturated or disrupted in advanced disease. This gradient, from null association in normal function, to significant inverse association in mild impairment, to absence of effect in severe CKD supports our central hypothesis: *H. pylori* influences uric acid metabolism through renal intestinal compensatory pathways that are active within a specific window of declining renal function.

Our findings carry several important implications. First, they suggest that the metabolic impact of *H. pylori* infection is modulated by renal function, underscoring the need for individualized consideration in both *H. pylori* eradication strategies and hyperuricemia management. In individuals with mild renal impairment, infection status may influence serum urate levels and should be integrated into metabolic risk assessments. Furthermore, future investigations into *H. pylori* associated metabolic effects should incorporate renal function stratification to avoid obscured associations and more accurately capture biological heterogeneity.

This study possesses several notable strengths. The large, community-based cohort enhances external validity, while standardized assessments of *H. pylori* status and renal function support measurement reliability. Importantly, this work introduces renal function as a novel and clinically meaningful effect modifier within the framework of host microbe metabolism interactions. Adjustment for a wide range of demographic, metabolic, and hepatorenal covariates further strengthens internal validity and mitigates confounding.

Nonetheless, several limitations should be considered when interpreting the findings. First, we did not assess strain level characteristics or bacterial load. *H. pylori* is genetically heterogeneous, with virulence factors such as CagA, VacA, and urease activity influencing host inflammatory responses and potentially affecting systemic metabolism (59–61). Without molecular characterization, we cannot determine whether specific bacterial phenotypes contributed to the observed associations. Second, the cross-sectional design precludes conclusions regarding causality or temporal direction. The 14C-UBT reflects current infection status but provides no insight into infection duration or cumulative exposure. Given the typically chronic nature of *H. pylori* colonization, infection duration may play a pivotal role in host metabolic adaptation (60). Prospective studies incorporating seroconversion data and serial uric acid measurements are needed to elucidate temporal dynamics. Third, although *H. pylori* is anatomically confined to the gastric mucosa, its systemic metabolic effects may be mediated through several established pathways, including low-grade systemic inflammation, altered gastric acidity affecting intestinal transporter expression, and modulation of the gut-kidney axis (34, 52, 62). While the effect size observed in this study was modest and unlikely to affect clinical thresholds for urate lowering therapy, the biological relevance remains noteworthy particularly in individuals with early renal dysfunction, where cumulative metabolic burden may evolve over time. Fourth, residual confounding cannot be excluded. Data on dietary purine intake, alcohol consumption, and use of uric acid modulating medications were not available. Endoscopic or histological markers of gastric pathology were also lacking, precluding correlation between mucosal inflammatory severity and systemic metabolic outcomes. Additional unmeasured variables such as prior *H. pylori* eradication, bacterial density, and host microbiome interactions may have influenced the findings. Finally, the subgroup with severe renal impairment (eGFR <60 mL/min/1.73 m^2^) was relatively small (*n* = 84) and exhibited a low prevalence of *H. pylori* infection (20.2%), limiting statistical power and resulting in wide confidence intervals. The reduced infection rate in this subgroup may itself have biological significance, as advanced chronic kidney disease is associated with elevated urea, altered gastric pH, and frequent antibiotic use factors that may inhibit *H. pylori* colonization. Moreover, the findings may not be generalizable to non-Chinese populations, in whom genetic variation in urate transporters and microbial susceptibility may differ.

In conclusion, this study identifies renal function as a key modifier of the association between *H. pylori* infection and serum uric acid levels. A significant inverse relationship was observed exclusively in individuals with mild renal impairment, likely reflecting synergistic interactions between *H. pylori* induced intestinal adaptations and compensatory urate excretion pathways. These findings provide mechanistic insight into the gut-kidney axis and illustrate how localized infections may exert systemic metabolic effects in the context of subclinical organ dysfunction. Although not yet practice changing, these results contribute to a growing framework in which host microbe interactions are interpreted through the lens of organ specific physiology. Given the inherent limitations of cross-sectional observational design, these findings should be regarded as hypothesis generating and warrant validation through prospective, mechanistic studies with longitudinal follow up.

## Conclusion

Our results suggest that serum uric acid levels in the Chinese population studied are influenced by *Helicobacter pylori* infection, with renal function appearing to modify this relationship. While an initial positive association was observed, this became non-significant after adjusting for confounders. Notably, an inverse association was found in individuals with mild renal impairment. These findings highlight the potential role of renal function in the relationship between *H. pylori* infection and uric acid metabolism. Further longitudinal studies with more detailed renal assessments and molecular characterization of *H. pylori* are needed to better understand the underlying mechanisms and temporal dynamics of this association.

## Data Availability

The original contributions presented in the study are included in the article/Supplementary material, further inquiries can be directed to the corresponding author.
