# Supplementary material for: Association between Helicobacter pylori infection and serum uric acid levels in a Chinese community population: a cross-sectional study stratified by renal function
Source: Front Med (Lausanne). 2025 Aug 29;12:1615161. doi: 10.3389/fmed.2025.1615161 (PMC12425895; doi:10.3389/fmed.2025.1615161)

Supplementary Table 1. Interaction analysis between H. pylori infection and potential effect modifiers on serum uric acid levels

Variable	Interaction Term Coefficient (β)	Standard Error	t-statistic	P-value	Interpretation
eGFR(per mL/min/1.73m²)	0.311	0.116	2.679	0.007	Significant modifier
ALT (per U/L)	-0.208	0.099	-2.109	0.035	Significant modifier
AST (per U/L)	-0.216	0.169	-1.278	0.201	NS
Total bilirubin (per µmol/L)	0.238	0.373	0.638	0.524	NS
BMI (per kg/m²)	-1.086	0.634	-1.715	0.086	NS
Fasting blood glucose (per mmol/L)	-0.786	1.549	-0.508	0.612	NS
HDL-C (per mmol/L)	7.632	6.746	1.131	0.258	NS
LDL-C (per mmol/L)	0.493	2.949	0.167	0.867	NS
Triglycerides (per mmol/L)	-0.087	1.280	-0.068	0.946	NS
Age (per year)	0.111	0.186	0.596	0.551	NS
Male sex (vs. female)	2.402	5.669	0.424	0.672	NS
MAP (per mmHg)	0.122	0.168	0.724	0.469	NS
Hypertension (yes vs. no)	-5.005	4.929	-1.015	0.310	NS
Diabetes (yes vs. no)	-5.291	6.393	-0.828	0.408	NS
Current drinking (vs. never)	-6.453	5.112	-1.262	0.207	NS
Former drinking (vs. never)	-8.387	5.983	-1.402	0.161	NS
Current smoking (vs. never)	5.351	5.105	1.048	0.295	NS
Former smoking (vs. never)	-4.776	6.001	-0.796	0.426	NS
Occupational intensity					
Moderate (vs. light)	7.058	7.619	0.926	0.354	NS
Heavy (vs. light)	4.351	7.623	0.571	0.568	NS
RBC ($\times 10^{12}$/L)	2.836	4.672	0.607	0.544	NS
WBC ($\times 10^{9}$/L)	0.554	1.357	0.408	0.683	NS
Platelet count ($\times 10^{9}$/L)	0.003	0.037	0.074	0.941	NS

eGFR, estimated glomerular filtration rate; ALT, alanine aminotransferase; AST, aspartate aminotransferase; BMI, body mass index; HDL-C, high-density lipoprotein cholesterol; LDL-C, low-density lipoprotein cholesterol; MAP, mean arterial pressure; RBC, red blood cell count; WBC, white blood cell count; NS, not significant.

Notes:

Interaction terms were calculated as the product of H. pylori infection status (determined by ^{14}C-urea breath test) and each potential modifier

Coefficients represent the additional change in serum uric acid (μmol/L) per unit change in the continuous variable (or presence vs. absence for categorical variables) when H. pylori infection is present

Models were adjusted for all main effects and the specific interaction term

Variables are grouped by physiological category and ordered by P-value within each category

Only eGFR and ALT showed statistically significant interactions, suggesting their role as effect modifiers in the H. pylori-uric acid relationship

Supplementary Table 2. Optimal cutpoint analysis for eGFR stratification in the association between H. pylori infection and serum uric acid levels

eGFR Cutpoint	Model 1[a]		Model 2[b]		Clinical Interpretation
(mL/min/1.73m²)	β coefficient	P-value	β coefficient	P-value	
50	-2.45	0.606	-1.88	0.768	Severe CKD threshold
55	-3.72	0.458	-3.91	0.457	Stage 3b CKD
60	-4.88	0.330	-5.02	0.307	Moderate CKD threshold
65	-1.26	0.871	-2.41	0.645	Mild-moderate CKD
70	-3.85	0.470	-4.72	0.390	Mild CKD
75	-7.94	0.069	-8.82	0.061	Mild renal impairment
80	-11.23	0.015	-12.67	0.006	Selected cutpoint[c]
85	-13.86	0.007	-15.21	0.002	Optimal statistical cutpoint[d]
90	-10.42	0.024	-11.38	0.014	Near-normal function

eGFR, estimated glomerular filtration rate; CKD, chronic kidney disease; CI, confidence interval.

Notes:

[a] Model 1: Adjusted for age and sex only

[b] Model 2: Fully adjusted model including age, sex, BMI, MAP, smoking, drinking, occupational intensity, hypertension, diabetes, lipid profiles (HDL-C, LDL-C, triglycerides), liver function (ALT, AST, total bilirubin), fasting glucose, and blood cell counts

[c] Primary analysis cutpoint selected based on clinical relevance and statistical significance

[d] Cutpoint with strongest statistical association in fully adjusted model

Supplementary Table 3. Multicollinearity diagnostics using Generalized Variance Inflation Factor (GVIF)

Variable	Initial Model	Final Model[a]	Clinical Decision
	GVIF	$GVIF^{(1/(2\times Df))}$	GVIF
Age (years)	1.504	1.226	1.482
Sex (male/female)	2.382	1.543	2.355
BMI (kg/m²)	1.547	1.244	1.544
eGFR (mL/min/1.73m²)	1.007	1.003	1.007
MAP (mmHg)	1.931	1.389	1.929
Hypertension (yes/no)	1.847	1.359	1.843

Diabetes (yes/no)	1.709	1.307	1.708
Total cholesterol (mmol/L)	21.341	4.620	—
HDL-C (mmol/L)	4.023	2.006	1.570
LDL-C (mmol/L)[b]	17.208	4.148	1.139
Triglycerides (mmol/L)	3.657	1.912	1.293
ALT (U/L)	2.934	1.713	2.930
AST (U/L)	2.614	1.617	2.579
Total bilirubin (μmol/L)	7.789	2.791	1.100
Direct bilirubin (μmol/L)	8.905	2.984	—
Fasting glucose (mmol/L)	1.700	1.304	1.698
RBC ($\times 10^{12}$/L)	1.665	1.290	1.662
WBC ($\times 10^{9}$/L)	1.302	1.141	1.301
Platelet count ($\times 10^{9}$/L)	1.258	1.122	1.257
Drinking[c]	1.770	1.153	1.769
Smoking[c]	1.699	1.142	1.699
Occupational intensity[c]	1.636	1.131	1.619
H. pylori infection	1.013	1.006	1.013

GVIF, Generalized Variance Inflation Factor; Df, degrees of freedom; BMI, body mass index; eGFR, estimated glomerular filtration rate; MAP, mean arterial pressure; HDL-C, high-density lipoprotein cholesterol; LDL-C, low-density lipoprotein cholesterol; ALT, alanine aminotransferase; AST, aspartate aminotransferase; RBC, red blood cell count; WBC, white blood cell count.

a: Final model after excluding variables with severe multicollinearity (GVIF > 10)

b: LDL-C showed acceptable GVIF after removing total cholesterol, as the collinearity was primarily due to the mathematical relationship between total cholesterol, LDL-C, and HDL-C

c: Categorical variables with Df = 2 (three-level factors); $GVIF^{(1/(2\times Df))}$provides a comparable measure across variables with different degrees of freedom

Supplementary Figure 1. Residuals versus Fitted Values

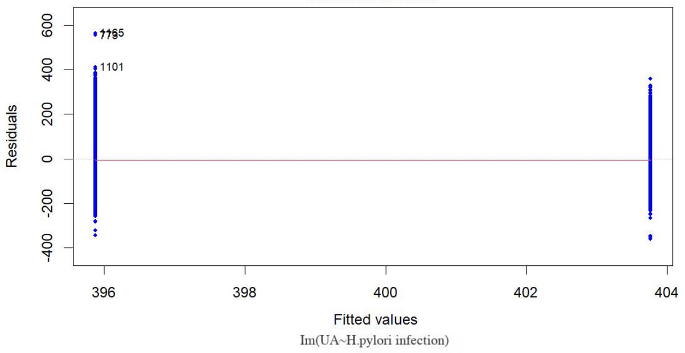

Supplementary Figure 2. Histogram of Residuals with Normal Curve Overlay

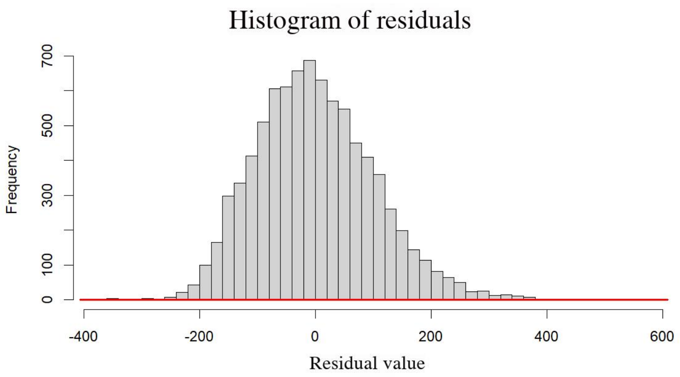

Supplementary Figure 3. Serum Uric Acid Levels by H. pylori Infection Status

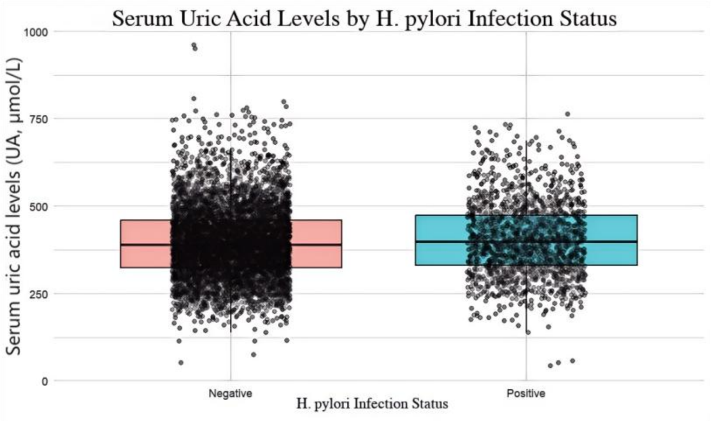

Supplementary Figure 4. Normal Q-Q Plot of Standardized Residuals

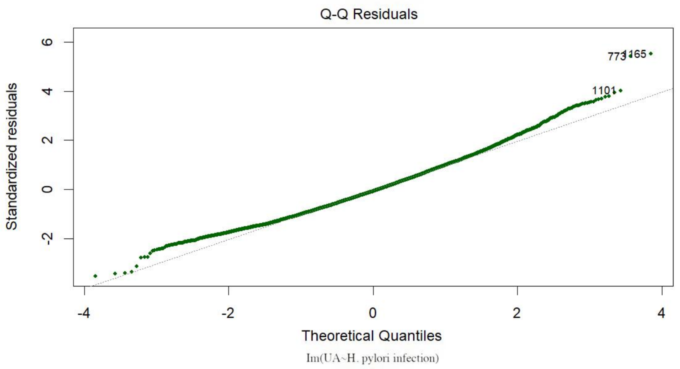

Supplement: Supplementary file 1 [file Data_Sheet_1.pdf]
